# Interatrial conduction disturbance in postoperative atrial fibrillation: a comparative study of P-wave dispersion and Doppler myocardial imaging in cardiac surgery

**DOI:** 10.1186/1749-8090-9-114

**Published:** 2014-06-24

**Authors:** Nima Hatam, Ali Aljalloud, Karl Mischke, Elias A Karfis, Rüdiger Autschbach, Rainer Hoffmann, Andreas Goetzenich

**Affiliations:** 1 Department of Thoracic and Cardio-Vascular Surgery, University Hospital RWTH Aachen, Pauwelsstr 30, D-52074 Aachen, Germany; 2 Department of Cardiology, Pulmonology, Angiology and Conservative Intensive Care, University Hospital RWTH Aachen, Pauwelsstr 30, D-52074 Aachen, Germany

**Keywords:** Electrophysiology, Echocardiography, Atrial fibrillation, CABG

## Abstract

**Objective:**

Disturbances of interatrial conduction have been proposed as one of the contributing mechanisms of postoperative atrial fibrillation (AF). P-wave dispersion has been recognized as a sensitive tool for detecting interatrial conduction disturbances. Doppler myocardial imaging (DMI) has been validated as a non-invasive tool to indirectly reflect electrical atrial activation and therefore is used in this study to detect possible interatrial electromechanical disturbances after cardiac surgery.

**Methods:**

30 patients (23 men, age 62 ± 1 years) admitted for coronary bypass surgery with no prior history of AF were included in this investigation. Echocardiography and electrocardiograms (ECG) were obtained on the day before and after surgery. In addition to standard echocardiography, DMI-loops were acquired from the apical window. The following time intervals were derived off-line from the free right atrial (RA), left atrial (LA) lateral and LA posterior wall: onset P-wave to start (P to A’start), to peak (P to A’peak) and to end of atrial deformation (total electromechanical activity). These intervals were compared to each other and to P-wave dispersion derived from the recorded ECGs.

**Results:**

All patients were in sinus rhythm during their postoperative assessment, but 11 patients presented episodes of AF within the first three postoperative days. Atrial electromechanical activation was earliest in the RA and latest in the lateral LA. In patients with AF, P-wave dispersion was significantly prolonged postoperatively (mean: +18.6 ms; 95% confidence interval (CI): 12.1–25.2 ms; p < 0.001) compared to non-AF patients (mean: -2.4 ms; CI: -6.6–1.9 ms). P dispersion was closely correlated to P to A’start intervals (from RA to LA lat.: preop.: rho = 0.74, postop.: rho = 0.87; p < 0.001). Prolonged right to left conduction interval was associated with an elevated risk for AF (from RA to LA lat.: odds ratio 1.13 (CI:1.03-1.24); p: 0.007.

**Conclusion:**

DMI enabled detection of interatrial conduction disturbances in concordance to findings of prolonged postoperative P-wave dispersion. Equally effective to P-wave dispersion, this simple and reproducible tool might help to early identify the risk for postoperative AF, thus extending the informative value of routine postoperative echocardiography.

## Background

Atrial fibrillation (AF) remains a common phenomenon in patients undergoing cardiac surgery, and is associated with increased morbidity and longer hospital stays [1-3]. Among multifactorial causes, interatrial conduction disturbances (IACD) have been repeatedly shown to be a trigger of paroxysmal AF in invasive electrophysiologic and non-invasive studies in non-surgical patients [4,5]. Furthermore IACD have been proposed as one of the contributing mechanisms of postoperative AF [6-8]. One of the most frequently reported methods to detect IACD non-invasively is P-wave dispersion in surface multichannel electrocardiograms (ECG) [6,8,9]. It represents a sensitive marker for IACD, but does not reveal changes and differences in electromechanical activation of the respective atrium. In modern ultrasound machines, Doppler myocardial imaging (DMI) has been demonstrated as a useful, non-invasive tool to indirectly reflect the brief moments of atrial electromechanical activation due to superb temporal resolution. Findings of DMI-derived atrial electromechanical activation in healthy individuals correlate well with invasive conduction dispersion [10,11].

The aim of this investigation was to assess the use of DMI for atrial electromechanical activation before and after cardiac surgery, and to evaluate its use as a possible predictor of postoperative AF.

## Methods

### Population

This investigation was approved by the local ethics committee of the RWTH Aachen University (EK 228/12). After giving their written consent, 30 patients (23 men) with a mean age of 62 ± 10 years with isolated coronary artery disease with no prior history of AF who were referred to our department for isolated coronary artery bypass grafting (CABG). Patients with (bi-)atrial dilatation (left atrial (LA) length ≤ 4.5 cm, LA Area ≤ 20 cm^2^) and/or impaired left ventricular systolic function (ejection fraction (EF) ≤ 50%) were excluded. Requirement of atriotomy (i.e. mitral valve repair), atrioventricular or intra-ventricular conduction disturbances and (corrected) congenital cardiac malformations were regarded as further exclusion criteria. Cardio-pulmonary bypass (CPB) was deployed in all patients using Bretschneider’s crystalloid cardioplegic solution for myocardial protection.

### Electrocardiography

12-channel Electrocardiogram (ECG) recorded on the day before surgery and on the first postoperative day were considered for this investigation [8]. P-wave dispersion, defined as the difference between the minimum and maximum P-wave duration in milliseconds, was measured in every recording [6]. Every patient’s ECG was continuously monitored after surgery for at least 5 days and AF events recorded by the attending staff. All ECG data were stored for 48 hours and reviewed on a daily basis. AF was defined as atrial arrhythmia persisting more than one minute.

### Echocardiography

**Figure 1:**
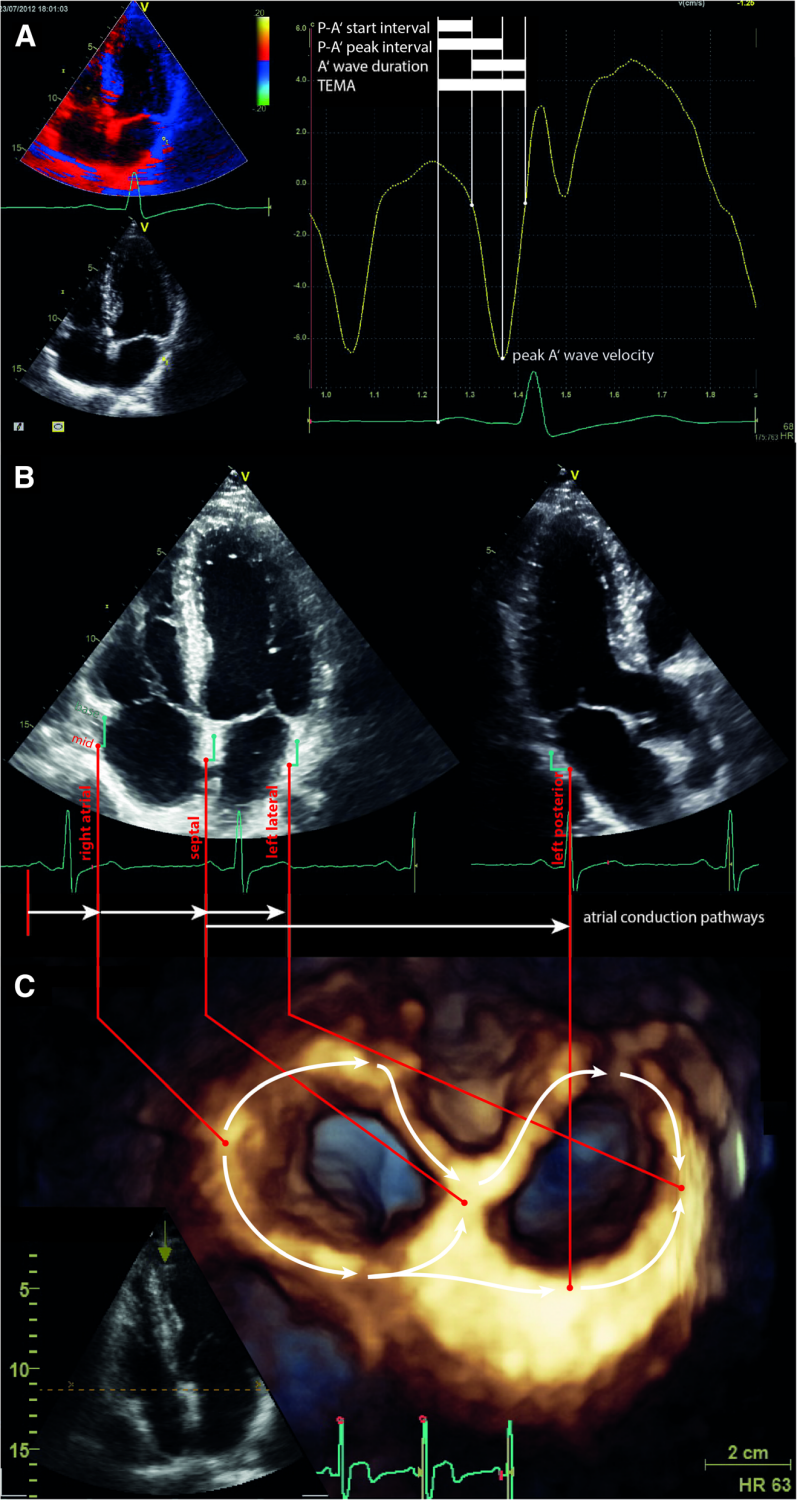
**Illustration of measured values on the basis of echocardiographic views. A**: Regions of interest (2×2 mm sample volume) placed at the RA, IAS, lateral LA and posterior LA studied at base and mid levels excluding the A-V-plane). **B**: time intervals measured in milliseconds: onset P-wave to start of atrial deformation (P to A’start), onset P-wave to peak of atrial deformation (P to A’peak) and onset P-wave to end of atrial deformation (total electromechanical activity, TEMA). Peak A-wave velocity measured in cm/s. **C**: Three-dimensional reconstruction of the atria, perspective from the apex towards the base of the heart (compare inset at lower left side). The white arrows indicate simplified atrial conduction pathways starting at the RA (figure’s left side), via the interatrial septum towards lateral LA (figure’s right side) and posterior LA (figure’s lower right side).

Standard two-dimensional (2-D) and Doppler-echocardiograms were digitally recorded using a Vivid E 9 ultrasound machine (Vivid E 9™, GE Vingmed, Horton, Norway) with a 2.5-5 MHz matrix phased array transducer (M5S®, GE Vingmed, Horton, Norway). Image sequences were acquired in parasternal (long/short axis), apical (four-, two- and three-chamber view) as well as subcostal (short/long axis) acoustical windows in two consecutive cardiac cycles for off-line analysis with simultaneous ECG-recording in lead II [12]. Left ventricular systolic function was measured by biplane ejection fraction using the Simpson’s method according to international recommendations [12]. Right ventricular longitudinal systolic function was measured by tricuspid annular plane systolic excursion (TAPSE [mm]).In the included patients, additional DMI-loops were recorded in four consecutive cardiac cycles in apnea from the apical acoustic window with frame rates above 200/s. The DMI data was studied off-line using EchoPac®’s Q-analysis tool (software version BT 12, GE Vingmed, Horton, Norway). The different atrial walls were identified from the apical views as follows: the right atrial wall (RA), the interatrial septum (IAS), and the left atrial lateral wall (LA-Lat) from the apical four-chamber view; the left atrial posterior wall (LA-Post) from the apical 3-chamber view (see Figure 1B). Each atrial wall was studied at base and mid levels, placing a 2x2 mm sample volume at respective atrial walls excluding the A-V plane verifying tracking of the sample volume during the entire cardiac cycle (see Figure 1A and B). The regional electromechanical function at each atrial wall was studied by the defined time intervals measured in milliseconds (see Figure 1A). From each atrial wall the tissue velocity profile obtained was used to measure the following parameters: onset P-wave to start of atrial deformation (P to A’start), onset P-wave to peak of atrial deformation (P to A’peak) and onset P-wave to end of atrial deformation (total electromechanical activity) (see Figure 1A). These time intervals were then compared between the atrial walls pre- and postoperatively. The difference of the time intervals of atrial electromechanical coupling (P to A’start) between the atrial walls was regarded as interatrial conduction time (see Figure 1B and C). Peak A’-wave velocities of the studied atrial walls were measured in cm/s.

### Statistical analysis

An independent professional statistician processed the presented data (p-werte.de, Jena, Germany). The precision of the derived parameters is expected to be around 2–5 ms. To increase accuracy of the presented data, all measurements were repeated in triplicate and means were subsequently calculated. Data were rounded to full milliseconds. Continuous variables are presented as mean ± standard deviation (SD).

Data were evaluated with and without adjustment for heart rate variability. The appropriate tests used were: linear regression analysis, Spearman’s ranking coefficient, logistic regression, *t*-test for paired data.

## Results

### Echocardiographic assessment of atrial conductance in cardiac surgery patients

All patients survived surgery without any serious adverse events. The results of left ventricular systolic function, right ventricular systolic longitudinal function and atrial sizes are summarized in Table 1.

**Table 1:** Pre and postoperative comparison of left ventricular systolic function, right ventricular systolic longitudinal function and atrial sizes

	**Preoperative**	**Postoperative**	**p-values**
**EF (%)**	55.0 ± 3.2	54.13 ± 2.73	n.s.
**TAPSE (mm)**	**22.73 ± 3.83**	**17.33 ± 2.92**	**<0.001**
**LA Area (cm**^**2**^**)**	17.06 ± 2.7	16.87 ± 2.32	n.s.
**LA Length (cm)**	4.16 ± 0.31	4.14 ± 0.28	n.s.
**RA Area (cm**^**2**^**)**	16.12 ± 2.55	16.09 ± 2.50	n.s.
**RA Length (cm)**	4.23 ± 0.33	4.25 ± 0.35	n.s.

**Bold** writing indicates significant values.

Postoperative left ventricular systolic function as well as atrial sizes did not differ to preoperative values. There was a significant postoperative decrease of right ventricular systolic longitudinal function measured by TAPSE.

The results of pre-and postoperative DMI-derived values are summarized in Table 2. Onset of electromechanical activation of the atria (P to A’start intervals) was earliest in the mid regions of the atria, starting with the RA followed by the IAS and left atrial walls respectively. Preoperatively, the differences of these intervals were longest between the RA and the lateral LA. In other words, interatrial conduction time was longest from the RA to the lateral LA (see also Figure 1B and C). There were no significant changes in postoperative interatrial conduction times in the whole patient cohort. However, subanalysis of the 11 patients presenting postoperative AF revealed a significant increase of interatrial conduction times between the base RA wall and base posterior LA wall (OR 0.95 (CI: 0.92-0.99), p: 0.017) and between the mid RA wall and mid lateral LA wall (OR 1.13 (CI: 1.03-1.24), p: 0.007).

**Table 2:** Pre- to postoperative comparison of conduction times between onset of P- wave and appointed atrial regions and locations

		***onset P wave to onset A’wave***	onset P wave to peak A’wave*	total electromechanical activity*
**Right atrium**	basal	-8 [-16;1] p = 0.092	**-29 [-44;-14] p < 0.001**	**-31 [-47;-15] p < 0.001**
	mid	-7 [-16;2] p = 0.105	**-27 [-42;-11] p = 0.001**	**-46 [-64;-28] p < 0.001**
**Lateral left atrium**	basal	-4 [-15;7] p = 0.517	**-14 [-27;-2] p = 0.023**	**-16 [-27;-6] p = 0.001**
	mid	-4 [-16;7] p = 0.092	**-21 [-31;-11] p < 0.001**	**-22 [-33;-11] p < 0.001**
**Interatrial septum**	basal	-7 [-16;2] p = 0.111	**-17 [-29;-5] p = 0.008**	**-19 [-32;-7] p = 0.003**
	mid	-7 [-15;1] p = 0.106	-13 [-27;1] p = 0.078	**-27 [-44; -9] p = 0.003**
**Posterior left atrium**	basal	-7 [-17;3] p = 0.177	-10 [-21;1] p = 0.067	-10 [-23;4] p = 0.159
	mid	-2 [-12;8] p = 0.717	**-13 [-25;0] p = 0.050**	**-12.5 [-24;-1] p = 0.039**

Negative differences indicate a faster postoperative atrial electromechanical cycle compared to its preoperative duration bold: significant values, *: heart rate adjusted. **Bold** writing indicates significant values.

According to the electromechanical activation, peak atrial longitudinal deformation, measured by P to A’peak intervals, was also earliest in the mid regions, starting with the RA followed by the IAS, posterior LA and latest in the lateral LA respectively (see also Table 3).

**Table 3:** DMI derived intervals in milliseconds

		**Preoperative**	**Postoperative**	**Difference**	
**Heart rate**		68 ± 12	84 ± 9	17 ± 14	
onset P wave to onset A’wave					
** RA**	base	41 ± 12	37 ± 11	-4 ± 15	
	mid	36 ± 11	32 ± 11	-4 ± 16	
** IAS**	base	57 ± 14	56 ± 15	-1 ± 14	
	mid	53 ± 16	52 ± 16	-1 ± 18	
** LA lat**	base	81 ± 15	82 ± 13	0 ± 18	
	mid	76 ± 16	76 ± 14	0 ± 21	
** LA post**	base	70 ± 18	68 ± 13	-2 ± 18	
	mid	62 ± 15	63 ± 15	1 ± 20	
onset P wave to peak A’wave					
** RA**	base	115 ± 22	85 ± 15	-30 ± 22	
	mid	108 ± 24	75 ± 15	-33 ± 27	
** IAS**	base	126 ± 22	104 ± 17	-21 ± 22	
	mid	117 ± 22	101 ± 19	-16 ± 19	
** LA lat**	base	137 ± 17	118 ± 18	-18 ± 21	
	mid	133 ± 18	108 ± 17	-25 ± 19	
** LA post**	base	126 ± 21	111 ± 19	-15 ± 22	
	mid	120 ± 20	104 ± 20	-16 ± 23	
Total electromechanical activity					
** RA**	base	186 ± 22	145 ± 26	-41 ± 31	
	mid	182 ± 21	130 ± 24	-52 ± 31	
** IAS**	base	193 ± 23	165 ± 17	-28 ± 20	
	mid	186 ± 23	154 ± 21	-32 ± 24	
** LA lat**	base	189 ± 19	171 ± 19	-18 ± 20	
	mid	188 ± 19	163 ± 27	-24 ± 22	
** LA post**	base	182 ± 25	164 ± 23	-18 ± 23	
	mid	173 ± 24	154 ± 18	-19 ± 23	

We observed a significant postoperative decrease of P to A’peak intervals within and between the atrial walls and regions, i.e. peak mechanical atrial longitudinal deformation occurred earlier postoperatively. In particular, peak atrial longitudinal deformation was reached earlier by far in the RA (RA base_pre-/postoperative_: 114.6 ± 21.8 ms vs. 84.9 ± 15 ms and RA mid_pre-/postoperative_: 107.7 ± 23.8 ms vs. 74.5 ± 14.6 ms; both p < 0.001).

Preoperatively, total electromechanical activation was comparable in all atrial regions and walls (180–190 ms; see Table 3). Postoperatively, a significant decrease in right atrial total electromechanical activity was noticed, whereas left atrial values remained fairly unchanged (RA-TEMA_preoperatively_: 186.2 ms ± 21.9 ms vs. RA-TEMA_postoperatively_: 145.2 ± 26.3 ms; OR 22.3 (CI: 36.3-10.3); p < 0.001).

Atrial peak longitudinal diastolic velocities were highest in the RA (base: -11 ± 3.1 cm/s, mid: -9.6 ± 3.2 cm/s, see also Table 4). The IAS and LA presented with similar values (exemplarily in base regions: IAS: -7.1 ± 1.5 cm/s, lateral LA: - 7.9 ± 1.9 cm/s, posterior LA: -7.5 ± 1.9 cm/s). Postoperatively, peak A’-wave velocities were significantly reduced in all atrial walls (exemplarily RA base: -11 ± 3.1 cm/s vs. -5.4 ± 1.9 cm/s; OR: -5.6(CI: -6.9 - -4.3;p < 0.001).

**Table 4:** Atrial peak longitudinal diastolic velocities (cm/s)

		**Preoperative**	**Postoperative**	**Difference**	**p-value**
**RA**	base	- 11 ± 3	- 5 ± 2	6 ± 3	<0.001
	mid	- 10 ± 3	- 4 ± 2	6 ± 3	<0.001
**IAS**	base	- 7 ± 2	- 6 ± 2	1 ± 2	<0.001
	mid	- 7 ± 2	- 5 ± 2	2 ± 2	<0.001
**LA lat**	base	- 8 ± 2	- 6 ± 2	2 ± 2	<0.001
	mid	- 8 ± 3	- 5 ± 2	3 ± 2	<0.001
**LA post**	base	- 8 ± 2	- 6 ± 2	2 ± 2	0.002
	mid	- 6 ± 2	- 4 ± 1	2 ± 3	<0.001

#### Influence of Heart Rate

We observed a significant increase in postoperative heart rate (67.67/min ± 11.8 vs. 84.3/min ± 9.8; p < 0.001). Due to this phenomenon we calculated the measured time intervals with and without heart rate adjustment in order to reveal a possible influence of the increased heart rate. However, heart rate adjustment did not alter the significance of pre- to postoperative comparison of the measured time intervals.

### Electrocardiography

#### P-wave dispersion and P-wave duration

All patients were in sinus rhythm during the 12-channel ECG-recording at the third postoperative day. Eleven patients had brief (<1 day) episodes of AF, recorded by continuous monitoring during the first three postoperative days. Mean heart rate was significantly higher postoperatively (67.7 ± 11.8/min vs. 84.3 ± 9.8/min; p < 0.001). No patient presented postoperative atrioventricular or inter-/intra-ventricular conduction disturbances (e.g. AV-Block, left bundle branch block etc.). Mean P-wave dispersion and duration was within normal ranges prior to surgery in the entire cohort with no significant postoperative changes (P-wave dispersion preoperative: 40.8 ± 8.2 ms vs. postoperative: 46.3 ± 12.6 ms; P mean duration: preoperative: 55.0 ± 3.2 vs. postoperative 54.1 ± 2.7; both n.s.). However, in eleven patients who presented temporary postoperative AF, a significantly prolonged P-wave dispersion was observed (P-wave dispersion_preoperative_: 40.5 ± 8.5 ms vs. P-wave dispersion_postoperative_: 59.5 ± 6.1 ms; p < 0.001).

#### Correlation of P wave dispersion to electromechanical coupling in patients suffering atrial fibrillation

In patients with episodes of AF, P-wave dispersion was significantly prolonged postoperatively (mean: +18.6 ms; 95% CI: 12.1–25.2 ms; p < 0.001) compared to non-AF patients (mean: -2.4 ms; CI: -6.6–1.9 ms). P dispersion was closely correlated to p to A’start intervals (from RA to LA lat.: p < 0.001, preop.: rho: 0.74, postop.: rho = 0.87; see Tables 5 and 6) and a prolonged right to left conduction interval was associated with an elevated risk for AF (from RA to LA lat.: odds ratio 1.13 (CI: 1.03-1.24); p:0.007)).

**Table 5:** Comparison of P-wave dispersion to conduction times by DMI (p start to A’wave intervals) in non-AF patient and AF patient cohorts

		**n**	**Mw**	**SD**	**OR**	**p-value**
From right atrial base	no AF	19	-5	14	1.23 (0.98 - 1.56)	0.076
to left atrial lateral base	AF	11	21	10		
From right atrial base	**no AF**	**19**	**-4**	**21**	**1.05 (1.01 - 1.09)**	**0.017**
to left atrial posterior base	**AF**	**11**	**14**	**17**		
From right atrial mid	**no AF**	**19**	**-6**	**20**	**1.13 (1.03 - 1.24)**	**0.007**
to left atrial lateral mid	**AF**	**11**	**21**	**13**		

**Bold** writing indicates significant values.

**Table 6:** Correlation of time intervals from electromechanical coupling derived by DMI to P-wave dispersion pre- and postoperatively in patients suffering from AF

	**Timepoint**	**P-wave dispersion**	
		**rho**	**p-value**
From right atrial base to left atrial lateral base	Preop.	**0.740**	**<0.001**
	Postop.	**0.870**	**<0.001**
From right atrial base to left atrial posterior base	Preop.	0.330	0.075
	Postop.	0.333	0.073
From right atrial mid to left atrial lateral mid	Preop.	**0.607**	**<0.001**
	Postop.	**0.786**	**<0.001**

**Bold** writing indicates significant values.

## Discussion

DMI presented a useful tool for the detection of atrial electromechanical coupling and hence indirect assessment of atrial electrical activation and interatrial conduction times. These DMI-derived values have been shown to reveal interatrial conduction disturbances (IACD) in patients presenting postoperative AF as shown by P-wave dispersion in surgical and invasive electrophysiology in non-surgical AF [4,8].

Proper tracking of the respective atrial wall with small regions of interest (ROI) as used in this study is very time-consuming taking up to 30 minutes and, after a learning curve of several exams, approximately 15 minutes. The chosen region of interest (e.g. base or mid portion) within the atrial wall did alter our measurements significantly. This coincides with the findings of Quintana et al., which showed that electromechanical intervals were longer near the atrio-ventricular ring than in the mid regions of the atria [13]. On the other hand, the qualitative implication of our measurements was unaltered by the numerical changes between base and mid atrial regions. Thus, in our opinion, the future use of DMI-derived measurements of atrial electromechanical coupling will not require small regions of interest, such as we used in our study. A size extension along the longitudinal axis of the ROIs was unproblematic (i.e. 6 mm), whereas ROIs should not be set closely to the AV ring and ROI-width should not exceed 2 mm in order to solely track atrial wall deformation. The measurement of all described values was very time-consuming as well, taking further 15 minutes per exam. For detecting IACD, the mere measurement of the respective P to A´start intervals, representing atrial electromechanical coupling, is sufficient enough. Therefore we used one large ROI and measured the intervals of electromechanical coupling only. This reduced each patient study to less than ten minutes making this technique useful for clinical application (data not shown).

Our findings of interatrial electromechanical coupling by DMI correlate well with findings of Quintana and Zhang, where electromechanical coupling was the earliest in the RA, followed by the IAS and the latest in the lateral LA [13,14]. There was no significant increase in postoperative interatrial electromechanical activation times in the entire cohort, but IACD were detected in 11 patients postoperatively who developed transient postoperative AF. An M-mode based DMI study by Omi and co-authors revealed that non-surgical patients with paroxysmal AF showed prolonged interatrial conduction times measured from the onset of P-wave to the beginning of tricuspid and mitral annulus backward motion [15]. Postoperative IACD was also detected by P-wave dispersion. In these patients, P-wave dispersion was significantly increased after surgery. Our findings compare well to the findings of Tsikouris and Koide who have shown that increased P-wave dispersion was associated with higher incidence of (postoperative) AF [8,9]. Changes in P-wave dispersion correlated well with differences in conduction time between the RA and LA derived by DMI. The fact that atrial mono- and/or dual-site pacing may prevent AF supports the concept of IACD [16-19]. In our study, IACD does not apply to every cardiac surgery patient, which seems to be one of the reasons why postoperative bi-atrial pacing is not effective in the prevention of postoperative AF in all patients [6,16]. In previous echocardiographic studies intraoperative transoesophageal echocardiography was used to assess IACD as measured by onset of P-wave to onset of left atrial appendage (LAA) systolic ejection as measured by pulsed-waved Doppler. The group found significant differences between pre- and immediate intraoperative measurements indirectly suggesting that open-heart surgery might lead to IACD. The measurement of the time interval between onset P-wave and LAA ejection flow does not offer the comparison of right and left atrial electromechanical activation. This method therefore cannot measure interatrial electromechanical coupling as comparing DMI-derived atrial activation with much higher temporal resolution, but represents a very easy and reproductive assessment tool for indirect detection of IACD and risk prediction of postoperative AF. Our data and the data from the Turkish group would strengthen the hypothesis of IACD being one of the major contributing factors of postoperative AF in a certain percentage of patients. The clinical implication of intra- and/or early postoperatively detected IACD would be bi-atrial pacing above intrinsic heart rate as recommended by the American College of Chest Physicians [20] and/or early commencement of anti-arrhythmic medication such as amiodarone [21]. In order to determine whether IACD is a homogenous disturbance of atrial conductive function or a localized abnormality by alteration of atrial conductive properties, further electrophysiologic and echocardiographic studies with larger numbers of patients and long-term follow-up are necessary.

Postoperative reduction of P to peak A’-wave intervals coincides with postoperative reduction of peak diastolic longitudinal deformation velocities, but not with TEMA. We noticed a significant postoperative reduction of right atrial TEMA while left atrial TEMA stayed fairly unchanged. Due to the fact that postoperative heart rates were significantly increased, we adjusted our measurements accordingly. This postoperative reduction of right atrial TEMA lead to a significantly increased difference between postoperative right and left atrial TEMA in the entire cohort. These findings might indicate decreased right atrial refractoriness as described in the literature by electrophysiologic studies [22]*.*

Three recent echocardiographic studies assessed the predictive value of pulsed-wave (PW)- DMI in patients prior to cardiac surgery [23-25]. Here, the time interval between onset of P-wave and peak of A’-wave derived from PW-DMI of the left lateral atrial wall was measured. This so called PA-TDI interval, if prolonged, correlated well to postoperative occurrence of AF as well as to fibrosis of the right atrial tissue [24]. Besides this, further methodical differences in comparison to our study have to be discussed. The scope of all three studies was the predictive value of the PA-TDI interval. Patients were therefore only assessed prior to surgery, not after, thus missing postoperative changes in electromechanical activation. The time interval from onset of P-wave to peak of A’-wave was regarded as representing atrial electromechanical coupling. There was no further explanation to this definition. As described by Quintana and in our opinion atrial electromechanical coupling is represented by the time interval of onset of P-wave to onset of A’-wave, when atrial deformation begins [13].

In all three studies frame-rates of PW-DMI were not described: Pre-settings in PW-DMI of the leading echocardiography machine vendors only show very low frame rates of approximately 35 frames per second, if the whole four chamber sector is used. Using 35 frames per second at a heart rate of 80/min means that 21 frames per cycle are recorded.

To increase frame rates the scanning sector must be reduced to the region of interest and frame rates per se increased on the respective machine setting in order to achieve frame rates higher than 90 frames per second. Looking at the figures of these studies, where in DMI-Mode the full apical four chamber view was used for pulsed-wave Doppler assessment, one must assume that frame rates were too low for measuring these brief electromechanical time intervals.

Nevertheless, PW-DMI with adequate frames rates measuring respective atrial electromechanical coupling (onset P-wave to onset A’-wave) should allow a more clinical application to detect interatrial conductance disturbances rather than carrying out the measurement off-line in quantitative analysis.

### Study limitations

The findings described in this study were obtained from a small group of patients. Larger consecutive studies might underline the postoperative phenomenon of interatrial conduction disturbance and shortened right atrial refractoriness. Furthermore, long-term follow-up has not been performed in this investigation where the development of atrial electromechanical (conduction time) and mechanical (peak systolic velocities) over time as well as the observation of heart rhythm in operated patients would be interesting.

From a methodological point of view, the surface ECG of ultrasound machines could be improved in order to identify the exact onset of P-wave due to the fact that the observation of atrial electromechanical activation takes place in just several milliseconds.

## Conclusion

DMI was able to detect disturbances in atrial electromechanical coupling in concordance to findings of prolonged postoperative P-wave dispersion. This simple, easy to obtain, reproducible, and clinically useful tool might help quantifying atrial electromechanical function, thus extending the informative value of routine postoperative echocardiography. Equally effective to P-wave dispersion, DMI derived atrial electromechanical activation may help to early identify the risk for postoperative AF.

DMI can be a helpful non-invasive tool to detect IACD. DMI might be a good predictor for postoperative AF. However, further investigations with larger numbers of patients are required to evaluate its value as a prediction tool.

## Abbreviations

AF: Atrial fibrillation; AV: Atrio-ventricular; CABG: Coronary artery bypass grafting; CI: Confidence interval; CPB: Cardio-pulmonary bypass; DMI: Doppler myocardial imaging; IACD: Interatrial conduction disturbance; IAS: Interatrial septum; ECG: Electrocardiography; LA: Left atrium; LAA: Left atrial appendage; OR: Odds ratio; PA-TDI: onset P-wave to peak A’wave of lateral LA (from 24); PW-DMI: Pulsed-wave Doppler myocardial imaging; RA: Right atrium; ROI: Region of interest; SD: Standard deviation; TAPSE: Tricuspid annular plane systolic excursion; TEMA: Total electromechanical activity; 2-D: Two-dimensional.

## Competing interests

The authors declare that they have no competing interests.

## Authors’ contribution

NH, AA and AG wrote the manuscript and interpreted statistics. NH and AA performed echocardiography and subsequent analysis. RH supervised study design and echo analysis. He also participated in the design of the study. NH, AA and EK recorded all ECGs and interpreted all monitor recordings. NH, AA and EK measured P-wave dispersion. KM supervised ECG interpretation and P-wave dispersion measurements. RA participated in the study design and coordination and helped to draft the manuscript. All authors read and approved the final manuscript.
